# Bloodstream Infection After Major Burn Surgery in Relation to Preoperative Glycemic Gap Among Patients Without Diabetes: A Retrospective Cohort Study

**DOI:** 10.3390/ijms27188137

**Published:** 2026-09-12

**Authors:** Jihion Yu, Young Joo Seo, In Suk Kwak, Young-Kug Kim

**Affiliations:** 1 Department of Anesthesiology and Pain Medicine, Asan Medical Center, University of Ulsan College of Medicine, Seoul 05505, Republic of Korea; jihionyu@amc.seoul.kr; 2 Department of Anesthesiology and Pain Medicine, Hallym University Hangang Sacred Heart Hospital, Seoul 07247, Republic of Korea; kwakisanes@gmail.com

**Keywords:** glycemic gap, bloodstream infection, burn injury

## Abstract

Major burn injury induces stress-related hyperglycemia, but absolute glucose concentrations do not account for chronic glycemic status. The glycemic gap, defined as the difference between measured glucose and hemoglobin A1c (HbA1c)-derived estimated average glucose, may reflect acute glycemic deviation from chronic glycemia. We investigated the association between preoperative glycemic gap and 30-day postoperative bloodstream infection (BSI) in adult burn intensive care unit patients without diabetes. In this retrospective cohort study, 352 patients undergoing burn surgery between 2014 and 2024 were included, of whom 217 (61.6%) developed postoperative BSI. Each 10-mg/dL increase in preoperative glycemic gap was independently associated with higher odds of BSI (odds ratio, 1.104; 95% confidence interval, 1.036–1.184; *p* = 0.003) after adjustment for clinical factors, including burn severity. This association remained consistent in a sensitivity analysis using the revised Baux score. Adding either preoperative glucose or glycemic gap to the clinical model improved model fit, whereas improvements in discrimination were modest, with no significant difference between the glucose and glycemic gap models. A greater preoperative glycemic gap was independently associated with subsequent BSI in patients with major burns without diabetes and may provide complementary information regarding acute glycemic disturbance.

## 1. Introduction

A major burn injury represents one of the most profound forms of metabolic stress in clinical medicine [1]. Extensive tissue damage triggers hypermetabolism, catecholamine surge, systemic inflammation, and marked insulin resistance, frequently resulting in stress-induced hyperglycemia even in patients without preexisting diabetes [2]. Dysregulated glucose metabolism in this setting has been associated with an increased risk of infection, organ dysfunction, and mortality [3,4]. Despite its clinical importance, interpretation and management of hyperglycemia in burn patients remain challenging. Absolute glucose levels are influenced by burn severity and inflammatory burden, and optimal glycemic targets in the burn intensive care unit (ICU) continue to be debated [3]. Intensive insulin therapy may reduce complications but increases the risk of hypoglycemia [5], whereas less intensive strategies may not fully characterize the magnitude of acute glucose elevation relative to an individual patient’s baseline glycemic status [6]. Therefore, measures that incorporate chronic glycemic exposure may provide complementary information to absolute glucose concentrations when evaluating stress-related dysglycemia [3,6].

The glycemic gap, defined as the difference between measured serum glucose and hemoglobin A1c (HbA1c)-estimated average glucose, has been proposed as a measure of acute glucose elevation relative to chronic glycemic status [7]. Previous studies have demonstrated associations between the glycemic gap and adverse outcomes in acute conditions, including ischemic stroke [8], ST-segment elevation myocardial infarction [9], and cardiogenic shock [10]. However, evidence regarding the clinical relevance of the glycemic gap in patients with major burn injury remains limited. This question may be particularly relevant in patients without previously recognized diabetes, in whom acute hyperglycemia during major burn injury may reflect a substantial departure from usual glycemic exposure [11].

Bloodstream infection (BSI) is a major infectious complication in patients with major burns and may develop during prolonged intensive care and repeated surgical treatment [12]. Although stress-related hyperglycemia has been associated with infectious complications in critically ill and burn populations [13,14], whether the preoperative glycemic gap is associated with subsequent BSI in burn patients without diabetes remains unclear. Therefore, this study aimed to evaluate the association between preoperative glycemic gap and 30-day postoperative BSI in adult burn ICU patients without previously diagnosed diabetes and with an HbA1c level < 6.5%. We additionally evaluated whether the glycemic gap provided incremental predictive information beyond clinical factors and absolute preoperative glucose concentrations.

## 2. Results

**Figure 1:**
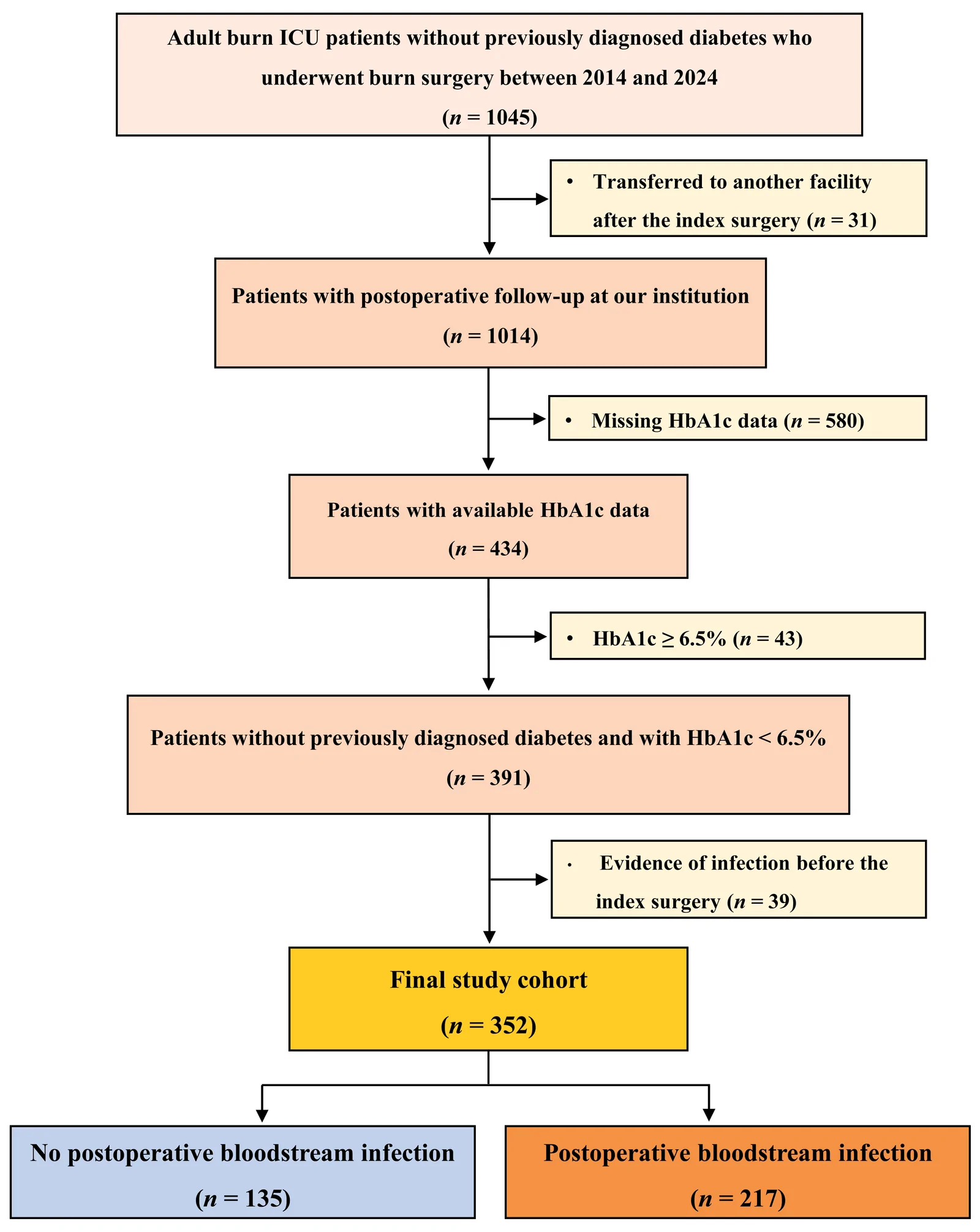
Flow diagram of patient selection for the study. Adult burn ICU patients without previously diagnosed diabetes who underwent burn surgery between 2014 and 2024 were screened for eligibility. Patients were excluded according to postoperative follow-up, HbA1c availability and level, and evidence of infection before the index surgery, as shown in the flow diagram. The index surgery was defined as the first burn-related surgical procedure performed after hospital admission. ICU, intensive care unit; HbA1c, hemoglobin A1c.

Among 1045 adult burn ICU patients without previously diagnosed diabetes who underwent burn surgery during the study period, 31 patients who were transferred to another facility after the index surgery were excluded because postoperative follow-up was unavailable at our institution. Of the remaining 1014 patients, 434 had available HbA1c measurements. After excluding 43 patients with HbA1c ≥ 6.5% and 39 with evidence of infection before the index surgery, 352 patients were included in the final analysis (Figure 1). Of these, 217 (61.6%) developed postoperative BSI, whereas 135 (38.4%) did not.

Baseline characteristics according to postoperative BSI status are presented in Table 1. Demographic characteristics were comparable between the groups. Patients who developed postoperative BSI had a higher prevalence of inhalation injury, greater total body surface area (TBSA) burned, and higher revised Baux scores (all *p* < 0.001). The burn mechanism also differed between the groups (*p* < 0.001). Preoperative glucose concentrations were higher in patients who developed postoperative BSI, whereas HbA1c levels were similar between the groups. Notably, the preoperative glycemic gap was greater in patients with postoperative BSI (39.2 [18.3–70.0] vs. 17.0 [−0.7–38.9] mg/dL, *p* < 0.001). Preoperative albumin concentrations were lower and lactate concentrations were higher in patients who developed postoperative BSI (both *p* < 0.001). Because HbA1c measurements were unavailable in a substantial proportion of potentially eligible patients, baseline characteristics were additionally compared according to HbA1c availability (Supplementary Table S1). Patients with available HbA1c measurements were older and had a higher prevalence of hypertension, and differences were also observed in TBSA burned, revised Baux score, lactate, and glucose.

Univariable and multivariable logistic regression analyses of factors associated with postoperative BSI are presented in Table 2. In the multivariable model including age, burn mechanism, inhalation injury, TBSA burned, albumin, lactate, and glycemic gap, a greater glycemic gap remained independently associated with postoperative BSI. Each 10-mg/dL increase in the glycemic gap was associated with a 10.4% increase in the odds of postoperative BSI (odds ratio, 1.104; 95% confidence interval, 1.036–1.184; *p* = 0.003). Inhalation injury (odds ratio, 2.189; 95% confidence interval, 1.281–3.789; *p* = 0.005) and greater TBSA burned (odds ratio, 1.056; 95% confidence interval, 1.037–1.078; *p* < 0.001) were also independently associated with postoperative BSI. In a sensitivity analysis in which age, TBSA burned, and inhalation injury were replaced by the revised Baux score, the association between preoperative glycemic gap and postoperative BSI remained consistent (odds ratio per 10 mg/dL, 1.109; 95% confidence interval, 1.041–1.189; *p* = 0.002) (Supplementary Table S2).

**Figure 2:**
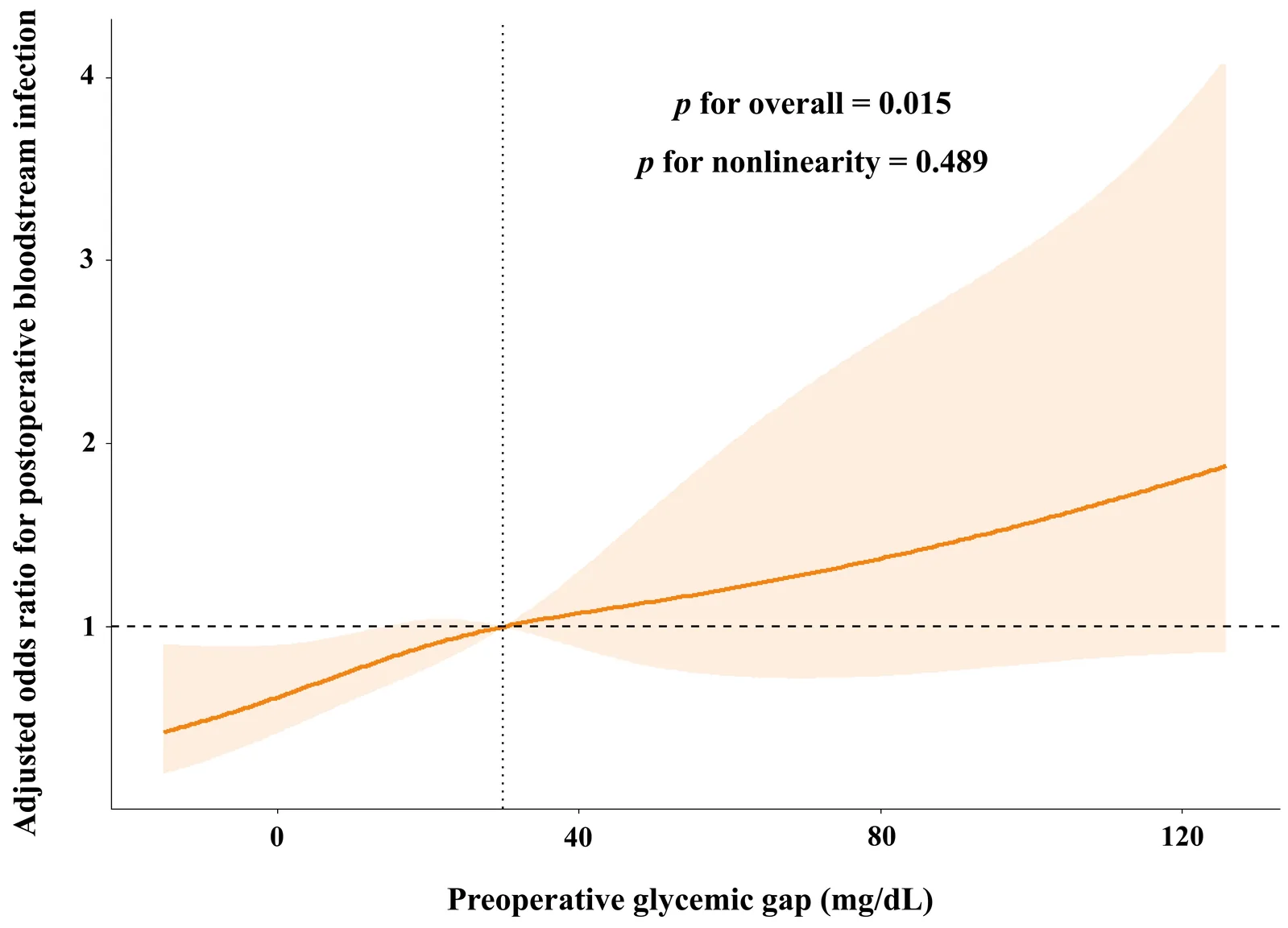
Restricted cubic spline analysis of the association between preoperative glycemic gap and postoperative bloodstream infection. The solid line represents the adjusted odds ratio, and the shaded area represents the 95% confidence interval. The restricted cubic spline model used four knots at the 5th, 35th, 65th, and 95th percentiles of the glycemic gap distribution (−15.0, 18.3, 46.9, and 125.9 mg/dL, respectively). The median glycemic gap (30.0 mg/dL) was used as the reference (odds ratio = 1), indicated by the vertical dotted line. The model was adjusted for age, burn mechanism, inhalation injury, total body surface area burned, albumin, and lactate. The overall association was significant (*p* = 0.015), with no evidence of nonlinearity (*p* = 0.489).

Restricted cubic spline analysis demonstrated a significant overall association between the preoperative glycemic gap and postoperative BSI (*p* for overall = 0.015), with no evidence of a nonlinear relationship (*p* for nonlinearity = 0.489) (Figure 2). The adjusted odds of postoperative BSI generally increased with increasing glycemic gap.

The incremental predictive performance of preoperative glucose and glycemic gap beyond the clinical model is presented in Table 3. Addition of either preoperative glucose or glycemic gap significantly improved model fit compared with the clinical model alone (likelihood-ratio test, *p* = 0.006 and *p* = 0.002, respectively). The area under the receiver operating characteristic curve (AUC) increased from 0.795 (95% confidence interval, 0.747–0.844) for the clinical model to 0.805 (95% confidence interval, 0.757–0.852) with preoperative glucose and 0.807 (95% confidence interval, 0.760–0.854) with glycemic gap; however, neither increase was statistically significant by the DeLong test (*p* = 0.217 and *p* = 0.171, respectively). The AUCs of the glucose and glycemic gap models also did not differ significantly (*p* = 0.380). Continuous net reclassification improvement was 0.293 (95% confidence interval, 0.097–0.499) for the glucose model and 0.301 (95% confidence interval, 0.088–0.496) for the glycemic gap model compared with the clinical model. In a direct comparison between the two extended models, the glycemic gap model yielded a continuous net reclassification improvement of 0.350 (95% confidence interval, 0.134–0.574) relative to the glucose model, whereas the difference in integrated discrimination improvement was not statistically significant (integrated discrimination improvement, 0.0051; *p* = 0.190). Bootstrap internal validation with 1000 resamples yielded optimism-corrected C-statistics of 0.782, 0.790, and 0.792 for the clinical, glucose, and glycemic gap models, respectively. The corresponding optimism-corrected calibration slopes were 0.930, 0.920, and 0.919, respectively.

## 3. Discussion

In this retrospective cohort study of adult patients with major burns without diabetes, a greater preoperative glycemic gap was independently associated with a higher risk of BSI within 30 days after the index surgery. This association persisted after adjustment for clinically relevant factors, with burn severity remaining strongly associated with BSI, whereas the associations with albumin and lactate were attenuated after multivariable adjustment. The association was also observed in a sensitivity analysis incorporating the revised Baux score. Restricted cubic spline analysis showed a generally increasing association between the glycemic gap and BSI risk, without evidence of significant nonlinearity. Adding either preoperative glucose or the glycemic gap to the clinical model improved model fit, whereas the improvements in discrimination were modest. Although the glycemic gap showed favorable continuous risk reclassification compared with preoperative glucose, the two models did not differ significantly in AUC or integrated discrimination improvement. Taken together, these findings suggest that the preoperative glycemic gap may provide additional information regarding subsequent BSI risk beyond established clinical factors and may capture aspects of acute glycemic disturbance not fully represented by absolute glucose levels; however, the present findings do not establish its predictive superiority over preoperative glucose.

Major burn injury induces profound hypermetabolic stress, catecholamine surge, systemic inflammation, and acute insulin resistance, resulting in stress-induced hyperglycemia even in patients without preexisting diabetes [2,5]. Hyperglycemia has long been associated with adverse outcomes after major burn injury, and previous burn-specific studies have also linked admission hyperglycemia to subsequent infectious complications. Notably, Ray et al. reported that admission hyperglycemia was independently associated with bacteremia and was also associated with pneumonia and urinary tract infection in burn patients [14]. These observations support the clinical relevance of acute glycemic disturbance in the burn population. However, absolute glucose concentrations do not distinguish acute stress-related elevation from an individual’s underlying chronic glycemic status. The glycemic gap attempts to account for this distinction by expressing the difference between the observed glucose concentration and the HbA1c-derived estimated average glucose, thereby representing the magnitude of acute glycemic deviation from estimated chronic glycemia [11].

Several indices have been proposed to characterize stress-related hyperglycemia by accounting for chronic glycemic status. Previous burn research has highlighted the potential importance of distinguishing acute from chronic hyperglycemia when evaluating outcomes after burn injury [15]. Among HbA1c-adjusted indices, the stress hyperglycemia ratio expresses acute glucose relative to HbA1c-derived estimated average glucose [16], whereas the glycemic gap expresses the corresponding deviation as an absolute difference [7]. In the present study, adding either preoperative glucose or the glycemic gap to the clinical model improved model fit, while the differences in discrimination between the glucose and glycemic gap models were modest and not statistically significant. Thus, the value of the glycemic gap in the present setting should not be interpreted as evidence of superior predictive performance over absolute glucose. Rather, the glycemic gap may provide a complementary perspective on acute glycemic disturbance by contextualizing the observed glucose concentration relative to an individual’s estimated chronic glycemic background.

The clinical significance of the glycemic gap has been investigated in various acute conditions, including ischemic stroke [8], ST-segment elevation myocardial infarction [9], and cardiogenic shock [10]. However, the magnitude and clinical implications of these associations have varied across disease entities. In some settings, the glycemic gap has been associated with mortality [17], whereas other studies have reported associations with cardiovascular or renal outcomes [9,18]. Moreover, associations between the glycemic gap and clinical outcomes have differed according to diabetic status, suggesting that the underlying metabolic context may influence its clinical significance. Given the distinct hypermetabolic and inflammatory response to major burn injury, findings from other acute illnesses may not be directly generalizable to this population. The present findings extend this literature to patients with major burns without diabetes by demonstrating an association between preoperative glycemic gap and subsequent postoperative BSI.

BSI is a major infectious complication in patients with major burns. Extensive disruption of the skin barrier, impaired host immune responses, invasive devices, repeated surgical procedures, prolonged intensive care, and exposure to broad-spectrum antimicrobial therapy contribute to the high susceptibility of these patients to BSI [19,20]. Reported BSI rates vary considerably according to burn severity, study population, and observation period. Previous studies have reported BSI in approximately 20–30% of broader burn ICU [20] or severe-burn cohorts, whereas rates exceeding 60% have been reported in exceptionally severe burn populations [21]. The 61.6% incidence observed in the present study is therefore high but should be interpreted in the context of a selected burn ICU population undergoing surgery, with relatively extensive burn injury and 30-day postoperative surveillance. Differences in patient severity, definitions of BSI, blood-culture practices, and observation periods preclude direct comparison of incidence across studies. Nevertheless, the substantial burden of BSI in this population underscores the potential clinical relevance of identifying perioperative factors associated with subsequent infection.

Hyperglycemic conditions may compromise innate antimicrobial defense through alterations in neutrophil functions, including chemotaxis, phagocytosis, and microbial killing, while also contributing to oxidative and inflammatory dysregulation and endothelial dysfunction [22,23]. In severe burns, these glucose-related effects may occur on a background of substantial burn-induced immune dysfunction, including altered neutrophil and macrophage responses, potentially increasing susceptibility to infection [24,25]. Experimental evidence further suggests that high glucose concentrations can alter neutrophil responses, including reactive oxygen species production and neutrophil extracellular trap formation [22,26]. However, these pathways were not directly evaluated in the present study and therefore remain biologically plausible rather than demonstrated mechanisms underlying the observed association. Accordingly, a greater glycemic gap may represent not only acute glucose elevation relative to chronic glycemic background but also a marker of systemic metabolic stress and host vulnerability. The observed association between glycemic gap and BSI should therefore not be interpreted as evidence of a causal effect of hyperglycemia on infection.

The temporal interpretation of the present findings also warrants consideration. The glycemic gap was calculated from a single preoperative glucose measurement obtained around the time of the index surgery, whereas BSI was assessed during the subsequent 30-day postoperative period. During this interval, patients with major burns may undergo repeated operations and experience substantial changes in wound status, nutritional support, antimicrobial therapy, hemodynamic condition, and glycemic control, all of which may influence subsequent infection risk. Therefore, the preoperative glycemic gap should not be regarded as a persistent metabolic exposure throughout the postoperative period. Rather, it may serve as a marker of the patient’s metabolic stress and host vulnerability at a clinically relevant perioperative time point. The observed association should therefore be interpreted as a temporal risk association rather than evidence that an elevated preoperative glycemic gap directly causes subsequent BSI.

In the present study, measures of burn severity, including inhalation injury and TBSA burned, as well as the glycemic gap, remained independently associated with postoperative BSI, whereas the associations of preoperative albumin and lactate were attenuated after multivariable adjustment. This attenuation may reflect the influence of adjustment for burn severity and other clinical factors and should not be interpreted as diminishing the established prognostic relevance of albumin or lactate in critically ill patients.

Several limitations of this study should be acknowledged. First, this was a retrospective, single-center study, and residual or unmeasured confounding cannot be excluded despite multivariable adjustment and sensitivity analyses. In particular, detailed time-varying information on insulin administration, nutritional support, vasopressor use, corticosteroid exposure, and other treatments that could affect perioperative glucose levels was not consistently available. Second, HbA1c was not routinely measured in all potentially eligible patients. Patients with and without available HbA1c measurements differed in several baseline characteristics, including age, burn severity, and admission glucose levels, raising the possibility of selection bias and limiting the generalizability of the findings. Third, although HbA1c was measured at admission before blood transfusion, its value may still be influenced by factors affecting erythrocyte turnover, such as anemia and renal dysfunction, which may introduce error into the estimated chronic glycemic level and consequently the calculated glycemic gap. Fourth, the glycemic gap was derived from a single preoperative glucose measurement and therefore does not capture glycemic variability or changes in metabolic status during the postoperative course. Fifth, although BSI provides a relatively objective microbiological endpoint, blood cultures were obtained according to clinical suspicion rather than through a standardized surveillance protocol. Differences in culture frequency and clinical decision-making may therefore have introduced detection bias, and the possibility of misclassification cannot be completely excluded despite predefined criteria for common skin commensals. Finally, the observational design establishes an association but cannot determine whether the glycemic gap itself contributes to the development of BSI or primarily reflects the severity of underlying metabolic stress. Prospective multicenter studies incorporating serial glucose measurements and more detailed metabolic and treatment-related data are needed to validate these findings and clarify the clinical role of the glycemic gap in patients with major burns.

## 4. Methods

### 4.1. Study Design and Patients

This retrospective cohort study was conducted at Hangang Sacred Heart Hospital, a tertiary university hospital affiliated with Hallym University. Adult (≥18 years) burn ICU patients who underwent burn surgery between 2014 and 2024 were screened for eligibility. Burn ICU admission followed our institutional criteria [27]: TBSA burned ≥ 20%; TBSA burned ≥ 10% in elderly patients (≥65 years); high-voltage electrical burns; burn injuries accompanied by significant trauma or medical comorbidities; or inhalation injury. The index surgery was defined as the first burn-related surgical procedure performed after hospital admission.

Patients with previously diagnosed diabetes based on their medical history were excluded. Among patients without previously diagnosed diabetes, those without available HbA1c measurements were excluded, as were patients with an admission HbA1c level ≥ 6.5% to minimize the inclusion of previously unrecognized diabetes. Patients transferred to another facility after the index surgery were excluded because postoperative follow-up was unavailable at our institution. Patients with evidence of infection before the index surgery were also excluded. Preoperative infection was defined as a documented infectious diagnosis, a positive microbiological culture interpreted as representing active infection, or clinical findings judged to represent infection rather than the inflammatory response attributable to the burn injury.

### 4.2. Ethical Consideration

This study was approved by the Institutional Review Board of Hangang Sacred Heart Hospital (IRB No. 2026-003; approved on 7 April 2026). The requirement for informed consent was waived due to the retrospective and observational nature of the study.

### 4.3. Data Collection

Clinical data were extracted from the institutional data warehouse of Hallym University Medical Center, which consolidates electronic medical records and laboratory databases. Variables retrieved included demographic characteristics, comorbidities (hypertension and end-stage renal disease), burn characteristics (post-burn day at index surgery, burn mechanism, inhalation injury, TBSA burned, and revised Baux score), and laboratory measurements. Inhalation injury was identified based on bronchoscopic evaluation in conjunction with clinical presentation and carboxyhemoglobin levels. The revised Baux score was calculated as age (years) + TBSA burned (%) + 17 × inhalation injury (0 = absent, 1 = present) [28].

HbA1c was measured at hospital admission before blood transfusion. Preoperative laboratory variables, including hemoglobin, albumin, lactate, and glucose, were obtained within 24 h before the index surgery. Estimated average glucose was calculated from HbA1c using the following formula: Estimated average glucose (mg/dL) = (28.7 × HbA1c [%]) − 46.7 [29]. The preoperative glycemic gap was defined as the difference between preoperative glucose and HbA1c-derived estimated average glucose and was expressed in mg/dL [7].

### 4.4. Outcome Definition

The primary outcome was postoperative BSI occurring within 30 days after the index surgery. BSI was defined as the isolation of a clinically significant bacterial or fungal pathogen from one or more blood cultures [19,30]. For common skin commensals, including coagulase-negative staphylococci, Corynebacterium spp., and Bacillus spp., isolation of the same organism from at least two separate blood cultures was required for the episode to be classified as a BSI. Positive blood cultures were reviewed to distinguish true BSI from contamination based on the isolated organism, microbiological findings, clinical course, and antimicrobial management, with infectious disease consultation considered when available.

### 4.5. Burn ICU and Perioperative Management

Patients received standardized multidisciplinary burn care in the ICU, including protocolized fluid resuscitation, early enteral nutrition, and routine wound management with topical antimicrobial therapy. Hemodynamic management and urine output targets were maintained according to institutional critical care protocols. Surgical management primarily consisted of excision and grafting, with escharotomy or fasciotomy performed when clinically indicated. General anesthesia, intraoperative fluid therapy, transfusion, and vasoactive support were administered according to institutional perioperative protocols.

### 4.6. Statistical Analyses

Continuous variables are presented as medians with interquartile ranges and were compared using the Wilcoxon rank-sum test. Categorical variables are presented as frequencies and percentages and were compared using Pearson’s chi-square test or Fisher’s exact test, as appropriate.

Univariable and multivariable logistic regression analyses were performed to evaluate the association between preoperative glycemic gap and postoperative BSI. Covariates in the primary multivariable model were selected a priori based on clinical relevance and included age, burn mechanism, inhalation injury, TBSA burned, albumin, and lactate. The glycemic gap was modeled as a continuous variable, with odds ratios reported per 10-mg/dL increase. Multicollinearity among covariates was assessed using variance inflation factors.

Restricted cubic spline analysis was performed to examine the shape of the association between preoperative glycemic gap and postoperative BSI. Four knots were placed at the 5th, 35th, 65th, and 95th percentiles of the glycemic gap distribution, and the median glycemic gap was used as the reference. The overall association and departure from linearity were evaluated using Wald tests.

To assess the incremental predictive information provided by glycemic gap, a clinical model including age, burn mechanism, inhalation injury, TBSA burned, albumin, and lactate was compared with models additionally including either preoperative glucose or glycemic gap. Model fit was compared using the Akaike information criterion and likelihood-ratio tests. Discrimination was assessed using the AUC with 95% confidence intervals, and differences in AUCs were evaluated using the DeLong test. Incremental predictive performance was further assessed using the integrated discrimination improvement and continuous net reclassification improvement. Confidence intervals for the continuous net reclassification improvement were estimated using 1000 bootstrap resamples.

Internal validation was performed using 1000 bootstrap resamples to estimate optimism-corrected C-statistics and calibration slopes. As a sensitivity analysis for adjustment of burn severity, the primary model was refitted by replacing age, TBSA burned, and inhalation injury with the revised Baux score. In addition, baseline characteristics were compared between patients with and without available HbA1c measurements to assess potential selection associated with HbA1c availability.

All statistical tests were two-sided, and a *p* value < 0.05 was considered statistically significant. Analyses were performed using R software (version 4.3.2; R Foundation for Statistical Computing, Vienna, Austria).

## 5. Conclusions

In patients with major burns without diabetes, a greater preoperative glycemic gap was independently associated with a higher risk of BSI within 30 days after the index surgery. The glycemic gap may provide complementary information on acute glycemic disturbance beyond established clinical factors, although its predictive performance was not significantly superior to that of preoperative glucose. These findings support the potential relevance of considering chronic glycemic background when interpreting perioperative hyperglycemia in patients with major burns. Prospective multicenter studies are warranted to validate these findings and further clarify the clinical role of the glycemic gap in this population.

## Figures and Tables

**Table 1:** Baseline characteristics according to postoperative bloodstream infection.

Variables	Total Patients (*n* = 352)	No Postoperative Bloodstream Infection (*n* = 135)	Postoperative Bloodstream Infection (*n* = 217)	*p*-Value
Demographic characteristics				
Age (years)	57.0 (47.0–65.0)	56.0 (46.0–67.0)	57.0 (47.0–64.0)	0.895
Sex (male)	287 (81.5%)	109 (80.7%)	178 (82.0%)	0.872
Body mass index (kg/m^2^)	23.4 (21.5–25.7)	23.6 (21.3–25.8)	23.3 (21.5–25.4)	0.402
Hypertension	101 (28.7%)	43 (31.9%)	58 (26.7%)	0.362
End-stage renal disease	13 (3.7%)	4 (3.0%)	9 (4.1%)	0.773
Burn characteristics				
Post-burn day at index surgery (days)	3.0 (2.0–4.0)	3.0 (2.0–5.0)	3.0 (2.0–4.0)	0.207
Burn mechanism				<0.001
Non-electrical burn	308 (87.5%)	107 (79.3%)	201 (92.6%)	
Electrical burn	44 (12.5%)	28 (20.7%)	16 (7.4%)	
Inhalation injury	150 (42.6%)	37 (27.4%)	113 (52.1%)	<0.001
TBSA burned (%)	32.0 (20.0–50.0)	22.0 (13.0–34.0)	41.0 (27.0–58.0)	<0.001
Revised Baux score	98.0 (83.5–115.0)	86.0 (69.0–99.0)	106.0 (92.0–121.0)	<0.001
Preoperative laboratory data				
Hemoglobin (g/dL)	14.0 (11.0–16.5)	13.7 (11.2–15.7)	14.2 (10.7–17.1)	0.089
Albumin (g/dL)	2.5 (2.1–2.9)	2.7 (2.4–3.0)	2.4 (1.9–2.8)	<0.001
Lactate (mmol/L)	2.3 (1.4–3.8)	2.0 (1.1–3.1)	2.6 (1.6–4.0)	<0.001
Glucose (mg/dL)	141.5 (121.0–174.0)	128.0 (109.0–156.0)	153.0 (127.0–187.0)	<0.001
HbA1c (%)	5.6 (5.3–5.9)	5.6 (5.3–5.9)	5.6 (5.3–5.8)	0.967
Glycemic gap (mg/dL)	30.0 (8.3–59.5)	17.0 (−0.7–38.9)	39.2 (18.3–70.0)	<0.001

Data are presented as median (interquartile range) or number (%), as appropriate. Continuous variables were compared using the Wilcoxon rank-sum test, while categorical variables were compared using Pearson’s chi-square test or Fisher’s exact test, as appropriate. The revised Baux score was calculated as age (years) + TBSA burned (%) + 17 × inhalation injury (0 = absent, 1 = present). The glycemic gap was defined as the difference between preoperative glucose and the estimated average glucose derived from HbA1c. HbA1c was measured at hospital admission before blood transfusion; preoperative laboratory variables were measured within 24 h before the index surgery. TBSA, total body surface area; HbA1c, hemoglobin A1c.

**Table 2:** Logistic regression analyses of factors associated with postoperative bloodstream infection.

Variables	Univariable Analysis		Multivariable Analysis	
	OR (95% CI)	*p*-Value	OR (95% CI)	*p*-Value
Age	0.996 (0.982–1.011)	0.628	1.016 (0.997–1.035)	0.101
Burn characteristics				
Burn mechanism				
Non-electrical burn	1.00		1.00	
Electrical burn	0.304 (0.155–0.580)	<0.001	0.914 (0.390–2.124)	0.834
Inhalation injury	2.878 (1.824–4.608)	<0.001	2.189 (1.281–3.789)	0.005
TBSA burned	1.059 (1.044–1.076)	<0.001	1.056 (1.037–1.078)	<0.001
Preoperative laboratory data				
Albumin	0.454 (0.323–0.626)	<0.001	0.891 (0.585–1.367)	0.594
Lactate	1.179 (1.047–1.338)	0.008	0.918 (0.792–1.067)	0.259
Glycemic gap, per 10 mg/dL	1.174 (1.104–1.254)	<0.001	1.104 (1.036–1.184)	0.003

Odds ratios for continuous variables represent the effect per 1-unit increase, except for glycemic gap, which was scaled per 10-mg/dL increase. Non-electrical burn was used as the reference category for burn mechanism. The glycemic gap was defined as the difference between preoperative glucose and the estimated average glucose derived from HbA1c. OR, odds ratio; CI, confidence interval; TBSA, total body surface area; HbA1c, hemoglobin A1c.

**Table 3:** Incremental predictive performance of preoperative glucose and glycemic gap for postoperative bloodstream infection.

Model	AIC	Likelihood-Ratio Test *p*-Value	AUC (95% CI)	DeLong Test *p*-Value
Clinical model	392.1		0.795 (0.747–0.844)	
Clinical model + preoperative glucose	386.6	0.006	0.805 (0.757–0.852)	0.217
Clinical model + glycemic gap	384.4	0.002	0.807 (0.760–0.854)	0.171

Likelihood-ratio and DeLong tests compare each extended model with the clinical model. The AUCs of the glucose and glycemic gap models did not differ significantly (DeLong *p* = 0.380). The clinical model included age, burn mechanism, inhalation injury, total body surface area burned, albumin, and lactate. AIC, Akaike information criterion; AUC, area under the receiver operating characteristic curve; CI, confidence interval.

## Data Availability

The data presented in this study are available on request from the corresponding author. The data are not publicly available due to ethical restrictions.

## Supplementary Materials

The following supporting information can be downloaded at: https://www.mdpi.com/article/10.3390/ijms27188137/s1, Table S1: Baseline characteristics according to HbA1c availability; Table S2: Sensitivity analysis of factors associated with postoperative bloodstream infection using the revised Baux score.

## Abbreviations

The following abbreviations are used in this manuscript:

HbA1c	Hemoglobin A1c
BSI	Bloodstream infection
ICU	Intensive care unit
TBSA	Total body surface area
AUC	Area under the receiver operating characteristic curve
