# Supplementary material for: Bloodstream Infection After Major Burn Surgery in Relation to Preoperative Glycemic Gap Among Patients Without Diabetes: A Retrospective Cohort Study
Source: Int J Mol Sci. 2026 Sep 12;27(18):8137. doi: 10.3390/ijms27188137 (PMC13607657; doi:10.3390/ijms27188137)
Supplement: Supplementary file 1 [file ijms-27-08137-s001.zip › Supplementary Table S1.pdf]

Supplementary Table S1. Baseline characteristics according to HbA1c availability

Variables	Total patients (n = 1014)	HbA1c unavailable (n = 580)	HbA1c available (n = 434)	P-value
Demographic characteristics				
Age (years)	53.0 (42.0–63.0)	49.0 (38.0–61.0)	57.0 (47.0–65.0)	< 0.001
Sex (male)	813 (80.2%)	464 (80.0%)	349 (80.4%)	0.933
Body mass index (kg/m²)	23.5 (21.5–25.7)	23.6 (21.5–25.5)	23.4 (21.4–25.9)	0.968
Hypertension	218 (21.5%)	104 (17.9%)	114 (26.3%)	0.002
End-stage renal disease	34 (3.4%)	18 (3.1%)	16 (3.7%)	0.738
Burn characteristics				
Post-burn day at index surgery (days)	3.0 (2.0–5.0)	3.0 (2.0–5.0)	3.0 (2.0–5.0)	0.179
Burn mechanism				0.684
Non-electrical burn	884 (87.2%)	503 (86.7%)	381 (87.8%)	
Electrical burn	130 (12.8%)	77 (13.3%)	53 (12.2%)	
Inhalation injury	430 (42.4%)	239 (41.2%)	191 (44.0%)	0.407
TBSA burned (%)	34.0 (20.0–50.0)	35.0 (22.0–52.0)	32.0 (20.0–49.0)	0.026
Revised Baux score	96.0 (79.0–116.0)	93.5 (75.0–115.5)	98.0 (84.0–116.0)	0.028
Preoperative laboratory data				
Hemoglobin (g/dL)	13.3 (10.7–16.1)	13.2 (10.9–15.8)	13.4 (10.5–16.3)	0.515
Albumin (g/dL)	2.5 (2.1–2.9)	2.5 (2.1–2.9)	2.5 (2.1–2.9)	0.665
Lactate (mmol/L)	2.4 (1.5–3.9)	2.5 (1.6–4.0)	2.3 (1.4–3.7)	0.021
Glucose (mg/dL)	138.0 (118.0–166.0)	135.0 (114.0–159.0)	143.0 (122.0–179.0)	< 0.001

Data are presented as median (interquartile range) or number (%), as appropriate. Continuous variables were compared using the Wilcoxon rank-sum test, and categorical variables using Pearson's chi-square test or Fisher's exact test, as appropriate. The revised Baux score was calculated as age (years) + TBSA burned (%) + 17 × inhalation injury (0 = absent, 1 = present). Preoperative laboratory variables were measured within 24 h before the index surgery. TBSA, total body surface area; HbA1c, hemoglobin A1c.
