# Supplementary material for: Bloodstream Infection After Major Burn Surgery in Relation to Preoperative Glycemic Gap Among Patients Without Diabetes: A Retrospective Cohort Study
Source: Int J Mol Sci. 2026 Sep 12;27(18):8137. doi: 10.3390/ijms27188137 (PMC13607657; doi:10.3390/ijms27188137)
Supplement: Supplementary file 1 [file ijms-27-08137-s001.zip › Supplementary Table S2.pdf]

Supplementary Table S2. Sensitivity analysis of factors associated with postoperative bloodstream infection using the revised Baux score

Variables	Univariable analysis		Multivariable analysis	
	OR (95% CI)	P-value	OR (95% CI)	P-value
Burn mechanism				
Non-electrical burn	1.00		1.00	
Electrical burn	0.304 (0.155–0.580)	< 0.001	0.989 (0.434–2.240)	0.978
Revised Baux score	1.046 (1.034–1.059)	< 0.001	1.039 (1.025–1.054)	< 0.001
Preoperative laboratory data				
Albumin	0.454 (0.323–0.626)	< 0.001	0.738 (0.495–1.098)	0.133
Lactate	1.179 (1.047–1.338)	0.008	1.005 (0.876–1.158)	0.947
Glycemic gap, per 10 mg/dL	1.174 (1.104–1.254)	< 0.001	1.109 (1.041–1.189)	0.002

In this sensitivity analysis, age, TBSA burned, and inhalation injury included as individual covariates in the primary multivariable model were replaced by the revised Baux score. The revised Baux score was calculated as age (years) + TBSA burned (%) + 17 × inhalation injury (0 = absent, 1 = present). Odds ratios for continuous variables represent the effect per 1-unit increase, except for glycemic gap, which was scaled per 10-mg/dL increase. Non-electrical burn was used as the reference category for burn mechanism. The glycemic gap was defined as the difference between preoperative glucose and the estimated average glucose derived from HbA1c. OR, odds ratio; CI, confidence interval; TBSA, total body surface area; HbA1c, hemoglobin A1c.
